# LncRNA MACC1-AS1 sponges multiple miRNAs and RNA-binding protein PTBP1

**DOI:** 10.1038/s41389-019-0182-7

**Published:** 2019-12-10

**Authors:** Xiaona Zhang, Yanchun Zhou, Shaoying Chen, Wei Li, Weibing Chen, Wei Gu

**Affiliations:** 1 Department of Pathophysiology, The Key Immunopathology Laboratory of Guangdong Province, Shantou University Medical College, Shantou, Guangdong Province 515041 China; 2 Guangdong Provincial Lab for Breast Cancer Diagnosis & Treatment, Shantou, China

**Keywords:** Cell growth, Breast cancer, Non-coding RNAs

## Abstract

Long noncoding RNA (lncRNA) represents a class of endogenous RNAs that regulate gene expression in eukaryotes. To date, the function and underlying mechanism of the majority of mammalian lncRNAs remain unknown. Here, we report that MACC1-AS1, a cognate antisense lncRNA of the sixth intron of the MACC1 gene, functions as a cell growth modulator and enhances breast tumor progress. RNA pulldown and luciferase assays showed that MACC1-AS1 contained binding sites for multiple miRNAs, including well-known tumor suppressors miR-384 and miR-145-3p that repress the expression of pleiotrophin (PTN) and c-Myc mRNAs. Binding of miR-384 and miR-145-3p miRNAs to MACC1-AS1 alters the cell growth phenotype through increased expression of PTN and c-Myc mRNAs. MACC1-AS1 also competitively interacted with PTBP1, an RNA-binding protein, via a conserved pyrimidine rich motif within this lncRNA. Binding of PTBP1to MACC1-AS1 not only stabilized MACC1-AS1 and enhanced the sponge effect of MACC1-AS1 on miRNAs, but also decreased PTBP1 availability for binding to target mRNAs. Our results define a new dimension into how a lncRNA is able to regulate cell growth by sponging multiple miRNAs and an RNA-binding protein.

## Introduction

Long noncoding RNAs (lncRNAs) belong to a group of abundant transcripts that are above 200 nucleotides in length and have no protein coding potential^1^. LncRNAs have been reported to play fundamental roles in the control of diverse cellular functions through transcriptional and post-transcriptional regulation of gene expression^2,3^. For example, HOTAIR^4^ and NEAT1^5^ are involved in reprograming chromatin status through recruiting chromatin-modifying complexes to modulate gene transcription. HULC and UCA1 have been shown to act as miRNA sponges competing with mRNA for miRNA binding^6,7^. In addition, GADD7 and OIP5-AS1 are implicated in controlling mRNA stability through interaction with RNA-binding proteins (RBPs)^8,9^. These functions allow lncRNAs to participate in a variety of physiological and pathological processes including cancer and other diseases^10,11^.

Current studies have revealed that post-transcriptional regulation of gene expression is basically controlled by two classes of regulatory molecules: RBPs and noncoding (ncRNAs)^12,13^. RBPs affect all aspects of post-transcriptional gene regulation, including RNA splicing, nuclear transport, decay, stability, and translation^14,15^. In contrast, among ncRNAs, miRNAs comprise a well-studied subgroup that functions primarily to control RNA stability and translation^16,17^, whereas role of lncRNA has not been fully investigated.

MACC1-AS1 is a cognate antisense RNA of the sixth intron of MACC1 mRNA (metastasis-associated in colon cancer-1). Several studies have indicated that MACC1, which is upregulated in a variety of tumors, could serve as an independent prognostic marker in certain cancer types. In addition, MACC1 has been demonstrated to be a transcriptional regulator of EMT and a master oncogene that enhances cell proliferation, invasion, and chemotherapy resistance^18,19^. A recent study showed that MACC1-AS1-mediated stabilization of MACC1 mRNA results in metabolic plasticity of gastric tumor^20^.

In this study, we investigate the underlying molecular mechanism that regulates MACC1-AS1-mediated cell proliferation and breast tumor progression by identifying its interacting molecules. Using a method that is based on the addition of MS2-hairpin loops to MACC1-AS1 RNA and a recombinant protein MBP-MCP, which comprises a maltose-binding domain (MBP) and a domain that recognizes the MS2 hairpins (MCP), we show that MACC1-AS1 can serve as a platform for multiple miRNAs. MACC1-AS1 could bind to multiple miRNAs, including miR-145-3p and miR-384, to modulate the expression of their target mRNAs, such as PTN (pleiotrophin) and c-Myc, respectively. We further show that PTBP1, an RBP, preferentially binds to MACC1-AS1 through a conserved pyrimidine rich motif. Binding of PTBP1 not only facilitates the stability of MACC1-AS1 and enhances the sponge effect of MACC1-AS1 on miRNAs, but also competes with PTBP1 target mRNAs for PTBP1 binding. Our results reveal that the two trans-acting regulators, PTBP1 and miRNA, coordinate for binding to lncRNA MACC1-AS1, and the balance of this interaction directly influences cellular phenotype.

## Materials and methods

### Reagents

Primary antibodies used in the study were PTBP1 (Abcam, USA, Cat# ab5642), PTN (Abcam, USA, #ab133517), AGO2 (MBL International, Japan, #RN005M), c-Myc (9E10, Thermo Fisher, USA) and GAPDH (Cell Signaling Technology, Cat#5174). HRP-conjugated anti-mouse and anti-rabbit secondary antibodies were purchased from Santa Cruz Biotechnology (USA). PCR primers were purchased from IGE Biotech (Guangzhou, China). MACC1-AS1, PTBP1 siRNAs and miRNA mimics were purchased from Gene pharma (Suzhou, China) and are list in Suppl. Table S1.

### Cell culture

HEK293T cells and breast cancer cell lines MDA-MB-231, MCF-7, BT549, and T47D were purchased from ATCC (USA). Cells were grown in DMEM medium supplemented with 10% FBS, 100 units of penicillin/ml and 100 mg of streptomycin/ml. Cells were incubated at 37 °C and 5% CO_2_ in a humidified chamber.

### Plasmid construction

Human MACC1-AS1 (639 bp) cDNA was amplified by RT-PCR from total RNA isolated from MDA-MB-231 cells using the primers listed in Suppl. Table S2. Amplified MACC1-AS1 was cloned into the pCIP2 lentivirus plasmid (pCIP2-MACC1-AS1) at the Not I and Bam HI sites. pCIP2-MACC1-AS1-MS2 was constructed by introducing a six-repeat MS2 hairpin structure into the Bam HI site of the pCIP2-MACC1-AS1 plasmid. Construction of MS2-conjugated full-length, truncated or mutant MACC1-AS1 plasmid was performed using PCR-directed subcloning methods. Primers used for making MACC1-AS1 mutants are as listed in Suppl. Table S2. To construct luciferase reporter genes, the DNA fragments of wild-type (WT) and mutant MACC1-AS1 fragments were cloned 3′ of the Renilla luciferase gene of the psiCHECK-2 (Promega) plasmid (denoted as pluc-MACC1-AS1 and pluc-MACC1-AS1-mutants). All constructs were verified by sequencing.

### Cell proliferation and invasion assays

Procedures for cell proliferation (MTT) and invasion (transwell) assays were performed as described previously^7^.

### Luciferase assays

Cells were transfected with individual psiCHECK-2 constructs. After transfection for 36 h, the cells were harvested for firefly/Renilla luciferase assays using the Dual-Luciferase Reporter Assay System (Promega, USA). Luciferase activities were normalized to the empty plasmid (pLuc).

### Transfection, lentivirus production, and infection

Sequences of miRNA mimics and siRNA used in the study are shown in Supplementary Table S3. Transfection of MACC1-AS1 constructs, miRNA mimics or siRNAs was conducted with Lipofectamine 2000 reagent (Invitrogen, USA) using the protocol recommended by the manufacturer. At 48–72 h after transfection, cells were collected and used for further analysis. Lentivirus was generated by co-transfecting HEK-293T cells with the lentiviral vector and packaging plasmids as previously described^21^. Supernatants were collected 48 h after transfection, filtered and concentrated using Lenti-X concentrator (Clontech, USA). Concentrated virus was used to infect cells immediately or stored at −80 °C for later usage. Stably infected cells were selected using 2–5 μg/ ml puromycin for about 2 weeks. Expression levels of infected genes were detected by RT-qPCR or western blots.

### RNA immunoprecipitation (RIP) assays

RIP experiments were performed as previously described^21^. AGO2-RIP or PTBP1-RIP assays were conducted in HEK-293T or MDA-MB-231 cells using AGO2 antibody-, PTBP1 antibody- or normal IgG-conjugated Protein A resin (Sigma-Aldrich, USA). Immunoprecipitates were first verified by western blotting using the corresponding antibodies. Total RNAs were extracted from individual precipitates by using a RNeasy MinElute Cleanup Kit (Qiagen) and reversely transcribed using PrimeScript RT Master Mix (TaKaRa, Japan). The abundance of tested RNAs or miRNAs in the precipitates was detected by RT-qPCR.

### miRNA and protein sequencing after MS2 pulldown assays

MS2 pulldown assays were performed as previously described using a recombinant fusion protein (MBP-MCP) that contains a maltose-binding domain (MBP) and a domain (MCP) that recognizes the MS2 hairpins^7^. Briefly, recombinant MBP-MCP-conjugated amylose resin (NEB, USA) was prepared at 4 °C. Cell lysates prepared from cells expressing MS2-tagged MACC1-AS1 or MACC1-AS1 mutants were incubated with MBP-MCP-coated amylose resin at 4 °C for 4 h in the presence of RNase and protease inhibitors. After extensive washing, bound MACC1-AS1-MS2 RNP complexes were eluted with 100 μl lysis buffer containing 20 mM maltose. Aliquots of the eluted materials were used for analyzing MACC1-AS1-associated protein(s) by western blotting. The remaining precipitates were used to extract RNA by TRIzol (Invitrogen). Where indicated, miRNAs associated with MACC1-AS1 in the extracts were analyzed by miRNA sequencing at the Guangzhou RiboBio Co. (China).

### FISH and immunofluorescence analysis

To detect the cellular distribution of MACC1-AS1, Cy3-labeled probes were synthesized for fluorescence in situ hybridization (FISH) and FISH assays were performed as previously described^7^.

### In vitro pulldown assay using synthesized MACC1-AS1-MS2 and MS spectrometry

MACC1-AS1-MS2 chimeric cDNA or MS2 cDNA were subcloned into plasmid pSP73 (Promega, USA) at the Xba I and Eco RI sites. After linearizing the plasmids with Eco RI, RNAs were in vitro transcribed. Transcribed RNAs were incubated with amylose resin that was coated with purified recombinant MBP-MCP for 1 h. After washing the RNA-coated resin three times with buffer (150 mM NaCl, 0.5% NP-40, 50 mM Tris pH 7.5), the resin was incubated with cytoplasmic extracts prepared from cultured MDA-MB-231 cells at 4 °C overnight with gentle rotation. Resin was washed with buffer #1 (150 mM NaCl, 0.5% NP-40, 50 mM Tris pH 7.5) followed by three time washings with buffer #2 (300 mM NaCl, 0.5% NP-40, 50 mM Tris pH 7.5). After the final wash and centrifugation, proteins bound to the conjugated RNAs were eluted by boiling for 5 min in SDS buffer. The eluted proteins were identified by mass spectrometry in Wininnovate Bio. Corporation (Shenzhen, China). MS spectrometry data have been deposited into the ProteomeXchange database as accession number of IPX0001686000.

### RNA extraction and qRT-PCR

Total RNA or RIP-precipitated RNA was extracted using TRIzol (Invitrogen, Cat# 15596026). cDNA was synthesized using a reverse transcription system from Invitrogen (Cat# 11904018). Quantitative real-time PCR (qRT-PCR) was performed using a SYBR Green PCR kit (Applied Biosystems, Cat# A25742). Primer sequences used for qRT-PCR are shown in Supplementary Table 2.

### Western blotting

Protein samples were subjected to sodium dodecyl sulfate polyacrylamide gel electrophoresis (SDS-PAGE). Separated proteins were transferred onto a nitrocellulose membrane and incubated with corresponding primary antibodies PTBP1 (1:1000), PTN (1:1000), GAPDH (1:2000) for 1 h followed by horseradish peroxidase-conjugated secondary antibodies (1:5000) for 30 min. Signals were detected using an ECL kit (Invitrogen, Cat# WP20005). For sequential immunoblotting experiments, the membranes were washed with Tris-buffered saline and treated with Western Blot Stripping Buffer (Thermo Scientific, USA) for 1 h. After washing and re-blocking, the membranes were incubated with other primary antibodies if necessary.

### Bioinformatics

The expression profiles of MACC1-AS1 in human tumor samples and paired normal tissues of human tumor were obtained from the GEPIA website (http://gepia.cancer-pku.cn). The abundance of MACC1-AS1 expression was profiled based on RNA-seq expression data sets.

### Statistical analysis

Statistical significance of experimental results compared to control values was analyzed using Student’s *t* test (two-tailed). A *P *<* *0.05* value indicates the experimental results to be statistically significant. For experiments with more than two groups, statistical significance was assessed by one-way ANOVA assays.

## Results

### MACC1-AS1 induces proliferation and tumorigenesis of breast cancer cells

**Fig. 1:**
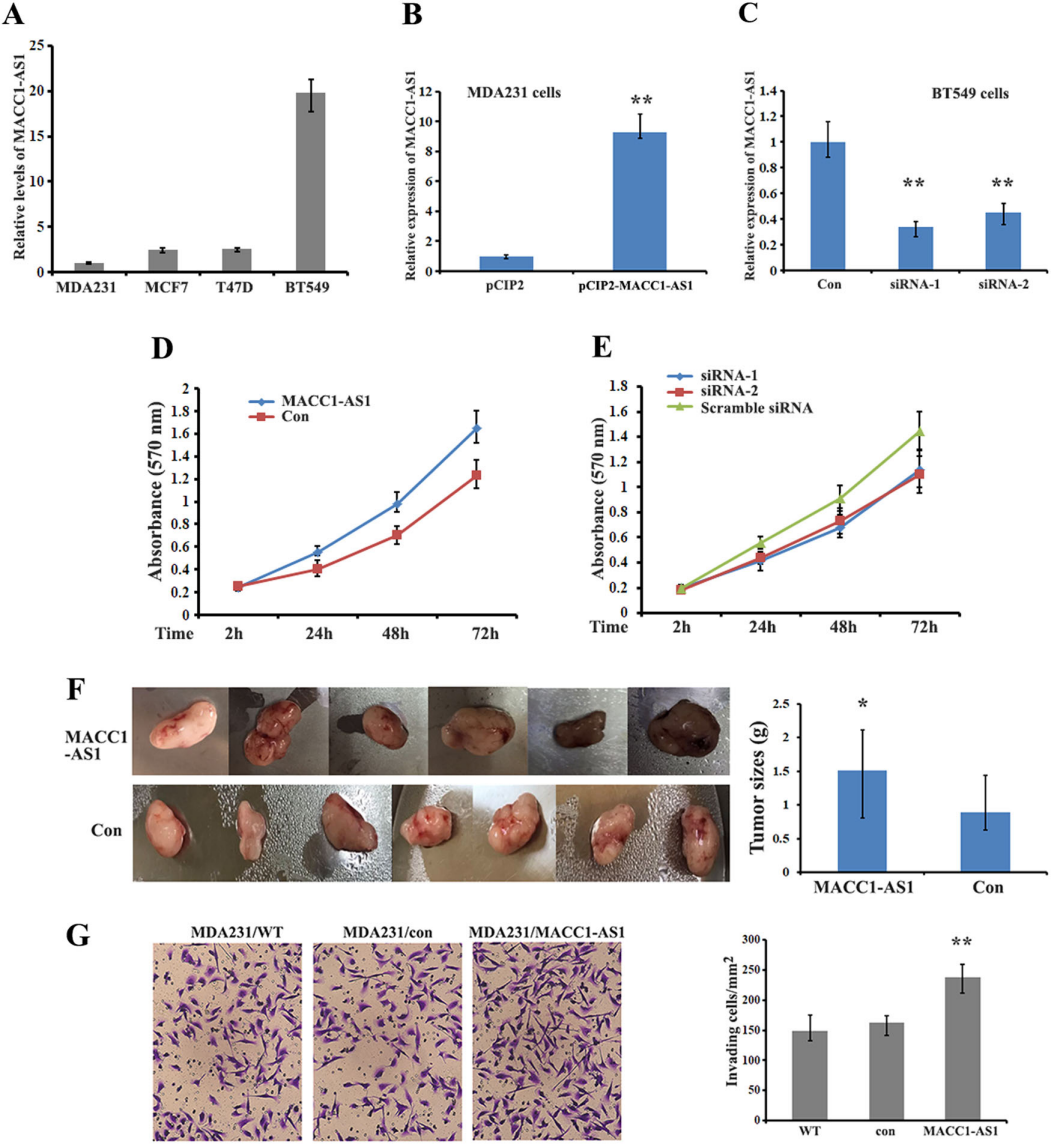
MACC1-AS1 induces breast carcinoma cell proliferation and tumorigenesis. **a** qRT-PCR showing the relative levels of MACC1-AS1 in breast cancer cell lines. **b**, **c** qRT-PCR results indicate the expression of MACC1-AS1 in MACC1-AS1-overexpressing (MDA-MB-231) and knockdown (BT546) cells. **d**, **e** MTT assays were used to measure proliferation in MACC1-AS1-overexpressing and -knockdown cells. *P *<* *0.01*. **f** Representative tumor samples and statistical analyses of tumor volumes in two xenograft groups are shown. Tumors derived from MDA-MB-231/MACC1-AS1 cells displayed larger primary tumor masses. *P *<* *0.05*. **g** Representative images and statistical analyses indicate the invasiveness of MACC1-AS1-overexpressing MDA-MB-231 and control cells. **P *<* *0.01*.

MACC1-AS1, a lncRNA without protein coding potential, is an antisense transcript within the sixth intron of primary MACC1 mRNA. Levels of MACC1-AS1 were detected in various breast cancer cell lines with the highest expression being observed in BT549 cells (Fig. 1a). RNA-seq data from the GEPIA database showed that MACC1-AS1 in various tumor tissues is not abundantly expressed (http://gepia.cancer-pku.cn/detail.php?gene=MACC1-AS1). To investigate the biological function of MACC1-AS1 in breast cancer cells, we established an MDA-MB-231 stable cell line overexpressing MACC1-AS1 and treated BT549 cells with siRNA to silence MACC1-AS1 expression (Fig. 1b, c). Cell proliferation assays (MTT) demonstrated that overexpression of MACC1-AS1 significantly increased, while knockdown of MACC1-AS1 expression by siRNA decreased cell propagation (Fig. 1d, e). Xenograft mice injected with MACC1-AS1 overexpressing MDA-MB-231 cells also exhibited considerably larger tumor volumes during the same observation period compared with the control group (Fig. 1f). In addition, transwell assays showed that cells overexpressing MACC1-AS1 had increased invasiveness compared to control and mock-transfected cells (Fig. 1g). These results indicate a pro-oncogenic role of MACC1-AS1 in breast cancer progression.

### Role of MACC1-AS1 on MACC1 mRNA expression

**Fig. 2:**
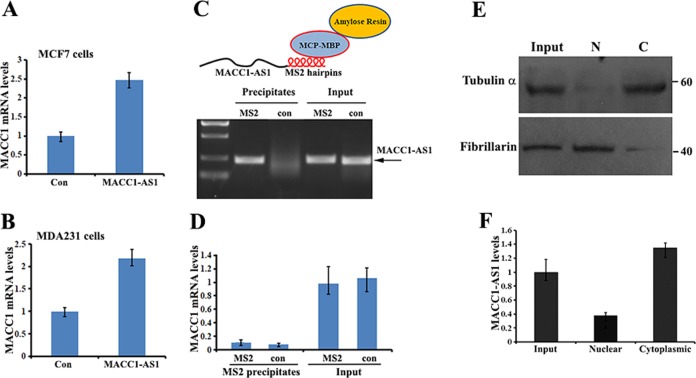
Role of MACC1-AS1 on MACC1 mRNA expression. **a**, **b** qRT-PCR showed that levels of MACC1 mRNA were increased in MACC1-AS1-overexpressing MCF7 and MDA-MB-231 cells. **c** Upper: schematic representation of the MACC1-AS1-MS2 chimeric RNA, which can be bound to amylose resin through recombinant protein MBP-MCP. Lower: RT-PCR and agarose gel electrophoresis showing that MS2-tagged MACC1-AS1 RNA was pulled down by MBP-MCP. **d** qRT-PCR showed that MACC1 mRNA was not detected in the precipitates of MACC1-AS1. **e** Representative western blots showing the expression of α-tubulin and fibrillarin in cytosolic (**c**) and nuclear (N) fractions of MACC1-AS1-overexpressing MDA-MB-231 cells. **f** qRT-PCR indicated that MACC1-AS1 is mainly present in the cytosolic fraction.

One of the functions of lncRNA is to bind antisense mRNAs and post-transcriptionally regulate their expression^22^. Since MACC1-AS1 is the cognate antisense RNA to the sixth intron of primary MACC1 mRNA, we initially suspected that the effect of MACC1-AS1 on promoting cancer cell progression could be through the post-transcriptional regulation of MACC1 mRNA. Indeed, levels of MACC1 mRNA were significantly increased in MCF7 and MDA-MB-231 cells overexpressing MACC1-AS1 (Fig. 2a, b). To determine whether this increase was resulted from the potential interaction of MACC1 mRNA with MACC1-AS1, we conjugated six MS2 hairpins into the 3′ of MACC1-AS1. We performed MS2 pulldown assays in lysates of MDA-MB-231 cells expressing MACC1-AS1-MS2 or MACC1-AS1 using a recombinant fusion protein (MBP-MCP) that contains a maltose-binding domain (MBP) and an MCP domain that recognizes the MS2 hairpins (Fig. 2c). qRT-PCR analysis indicated that neither MACC1 mRNA, nor the spliced sixth intron of primary MACC1 mRNA, co-precipitated with MACC1-AS1 (Fig. 2d, Suppl. Fig. S1A). C/N (cytoplasm and nuclear fractionation) and FISH (fluorescence in situ hybridization) assays showed that the MACC1-AS1 transcript was widely localized in the cell cytoplasm (Fig. 2e, f and Suppl. Fig. S1B). These results suggest that MACC1-AS1 indirectly affects MACC1 gene expression and could perform cytoplasmic functions.

### MACC1-AS1 serves as a platform for miRNA and Ago2 binding

**Fig. 3:**
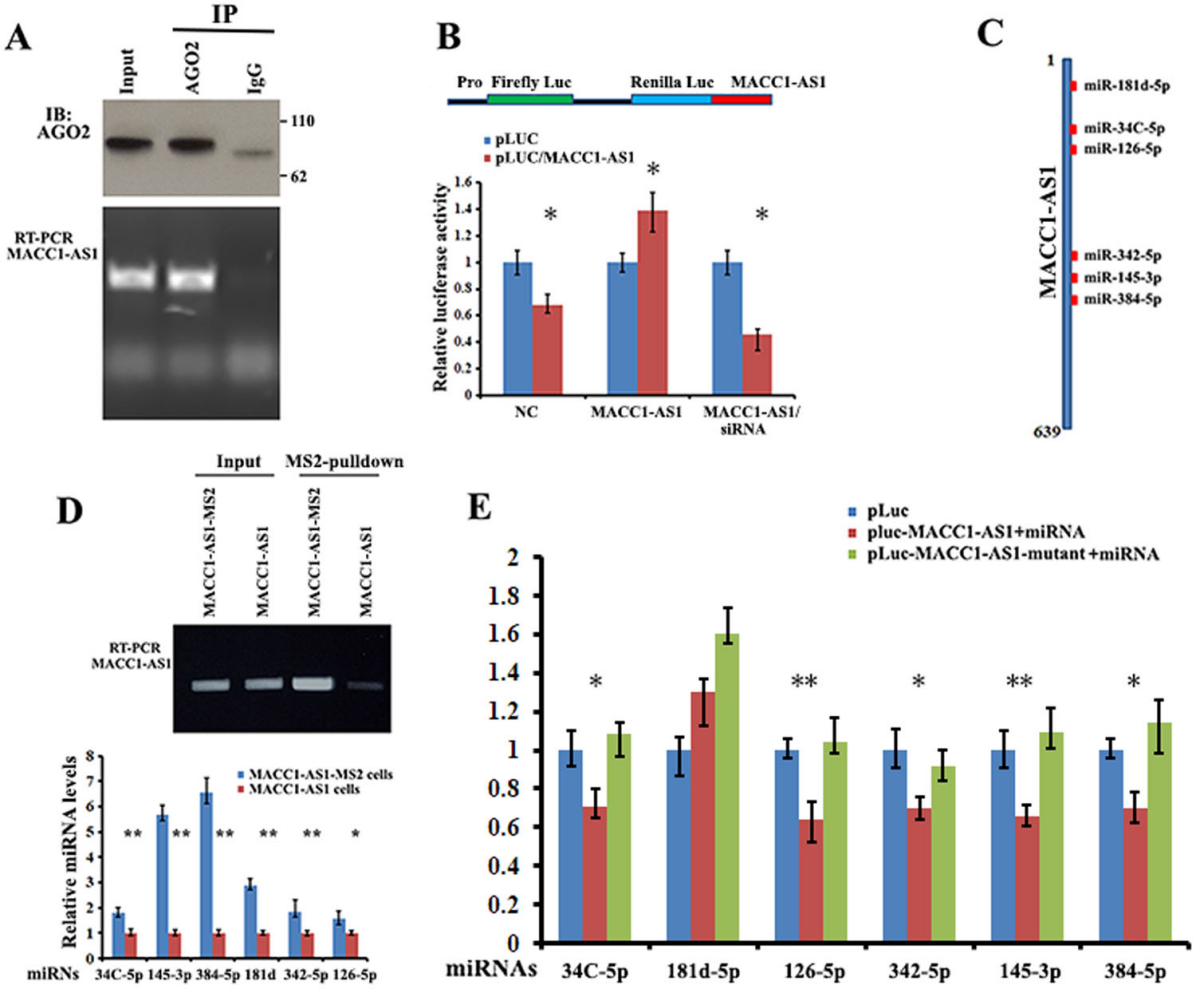
MACC1-AS1 serves as a platform for Ago2 and miRNA binding. **a** Upper: immunoprecipitation (IP) of Ago2 protein from extracts of HEK-293T cells. Lower: levels of MACC1-AS1 co-precipitated with Ago2 protein were analyzed by RT-PCR. **b** Upper: schematic of a luciferase reporter in which the entire MACC1-AS1 sequence was fused into the 3′ region of the Renilla luciferase gene of the psiCHECK-2 construct (denoted pLuc-MACC1-AS1). Lower: HEK-293T cells were transfected with pLuc (control) or pLuc-MACC1-AS1, or pLuc-MACC1-AS1 in combination with either the MACC1-AS1 expressing vector or siRNA against MACC1-AS1. Luciferase activities were determined after 36 h transfection using a dual-luciferase assay system. Renilla luciferase activity was normalized to the activity of firefly luciferase. *P *<* *0.05* as determined by Student’s *t* test. **c** Schematic representation of the potential binding sites for individual miRNAs within MACC1-AS1 RNA. **d** Upper: RT-PCR and agarose gel electrophoresis indicate that MACC1-AS1-MS2 was pulled down by MBP-MCP-conjugated amylose resin. Lower: individual miRNAs were detected by RT-qPCR in the precipitates of MACC1-AS1. **P *<* *0.01* as determined by Student’s *t* test. **e** HEK-293T cells were co-transfected with pLuc, wild-type or mutant MACC1-AS1 reporters with the corresponding miRNA mimics. Luciferase activity was determined by the dual luciferase reporter system. Activity of Renilla luciferase was normalized to the activity of firefly luciferase. **P *<* 0.01, *P *<* *0.05* as determined by one-way ANOVA followed by Tukey’s multiple comparison tests.

Given that lncRNAs can act as miRNA sponges and MACC1-AS1 is stably expressed in the cytoplasm, we investigate the potential of MACC1-AS1 to bind to miRNAs. We conducted RIP for Ago2 in HEK-293T cells and observed by RT–qPCR that endogenous MACC1-AS1 co-precipitated with Ago2 (Fig. 3a). We then constructed a dual luciferase reporter (psiCHECH-2, Promega) in which MACC1-AS1 was inserted downstream of the Renilla luciferase gene (pLuc/MACC1-AS1). We hypothesized that MACC1-AS1-associated miRNAs may potentially affect luciferase activity via miRNA-mediated activation of deadenylation and subsequent exonucleolytic degradation. This hypothesis was supported by the experiments that inclusion of MACC1-AS1 in the 3′UTR of Renilla luciferase causes downregulation of luciferase activity (Fig. 3b, NC). Moreover, either overexpressing MACC1-AS1 or knockdown of MACC1-AS1 by siRNA significantly changed Luc/MACC1-AS1 reporter activity (Fig. 3b, MACC1-AS1 or MACC1-AS1/siRNA, respectively), suggesting that MACC1-AS1 could serve as a platform for miRNA and Ago2 binding.

### Multiple miRNAs bind to MACC1-AS1

To identify the miRNAs that interact with MACC1-AS1, we analyzed potential miRNA binding sites within MACC1-AS1 RNA using the RNAhybrid prediction program and online bioinformatics tool (Krek, A; 2005; www.microRNA.org-target). This allowed us to identify MACC1-AS1 sequences complementary to the seed regions of six miRNAs, including miR-181d-5p, miR-34C-5p, miR-126-5p, miR-342-5p, miR-145-3p, and miR-384 (Fig. 3c and Suppl. Fig. S2A). To verify the binding ability of the predicted miRNAs to MACC1-AS1, we hereafter performed MS2 pulldown assays in lysates of MDA-MB-231 cells expressing MACC1-AS1-MS2 or MACC1-AS1 using amylose resin-conjugated recombinant fusion protein (MBP-MCP) that recognizes the MS2 hairpins (Fig. 2c upper). After incubating the resin with cell lysate, MACC1-AS1-MS2-bound amylose resin was precipitated and the potential of the six miRNAs that could be complexed with MACC1-AS1-MS2 were assayed by RT-qPCR. Results showed that two miRNAs, miR-145-3p and miR-384, were highly enriched in the precipitates, while another four miRNAs, miR-181d-5p, miR-34C-5p, miR-126-5p, and miR-342-5p, were also enriched at a relatively lower levels (Fig. 3d). To further assess the interactions between MACC1-AS1 and individual miRNAs, we mutated each putative miRNA binding site within the MACC1-AS1 sequence in the 3′UTR of the luciferase reporter (Suppl. Fig. S2B). After co-transfection of wild type or mutant reporters with the corresponding miRNA mimic into HEK-293T cells, we detected that with the exception of miR-181d-5p, five miRNAs were able to reduce the luciferase reporter activity by at least 30% (Fig. 3e, blue vs. red bars). However, transfection of individual mutant MACC1-AS1s lacking a given miRNA-binding site increased luciferase activity (Fig. 3e, red vs. green bars). Interestingly, transfection of the individual miRNAs into BT549 cells only slightly reduced cellular MACC1-AS1 levels (Suppl Fig. S3), suggesting that MACC1-AS1 could function as a miRNA sponge.

### Binding of miRNAs to MACC1-AS1 promotes cell proliferation

**Fig. 4:**
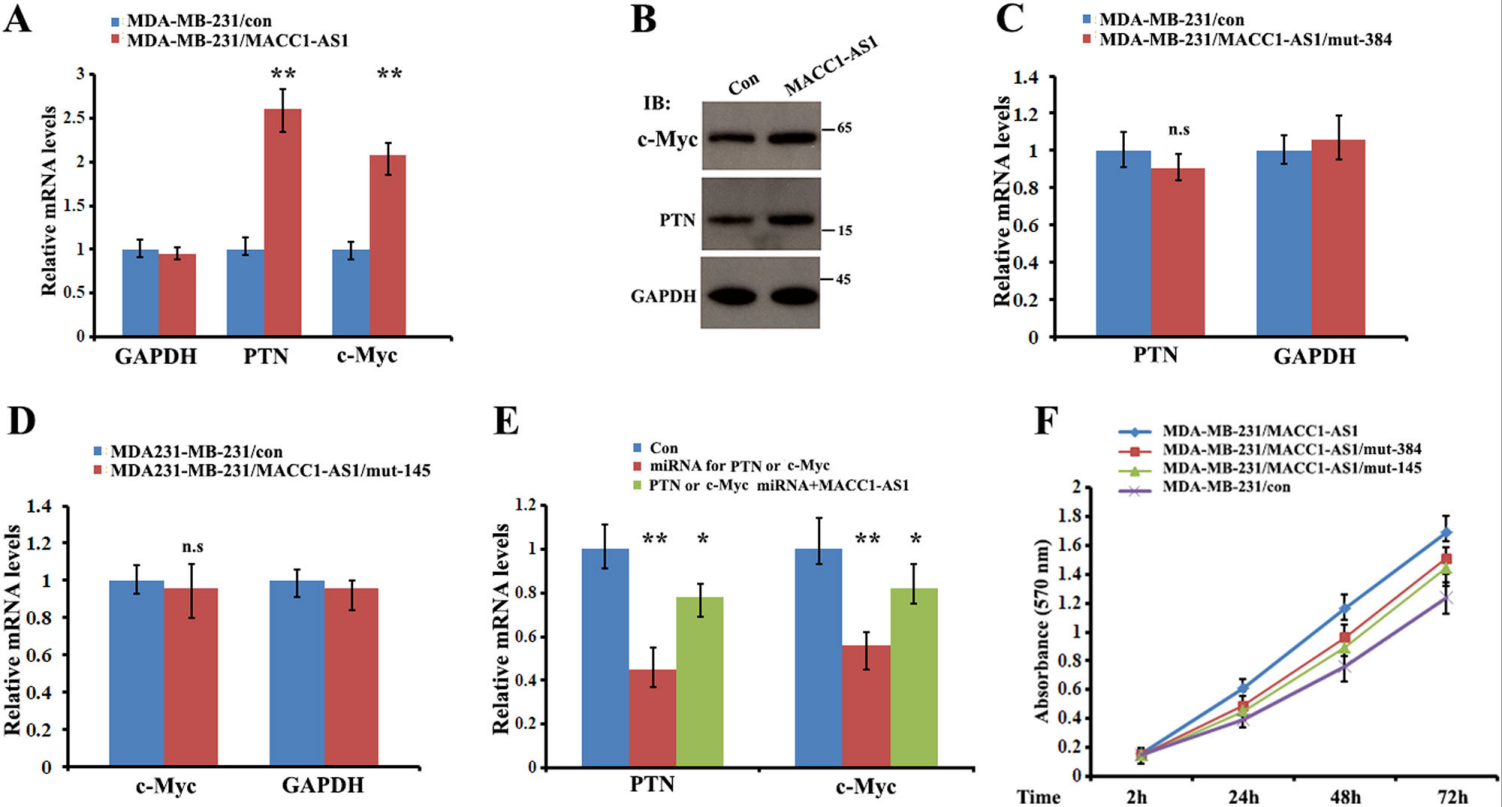
Binding of miRNAs to MACC1-AS1 promotes cell proliferation. **a**, **b** qRT-PCR and western blots indicate that cells overexpressing MACC1-AS1 increased the expression of PTN and c-Myc. **P *<* *0.01* as determined by Student’s *t* test. **c**, **d** qRT-PCR indicate that levels of PTN and c-Myc mRNAs were slightly changed when overexpressing MACC1-AS1 lacking a putative miRNA-384 or miRNA-145 binding site, respectively. n.s: not significant. **e** HEK-293T cells were transfected with miRNA-384 or miRNA-145 siRNA alone (red colored) or co-transfected with siRNAs and vectors expressing MACC1-AS1. qRT-PCR indicating that the miR-384-5p-mediated PTN or miR-145-3p-mediated c-Myc mRNA reduction could be partially rescued by overexpression of MACC1-AS1. **P *<* 0.01, *P *<* *0.05* as determined by one-way ANOVA assays. **f** MTT assays demonstrate that cells expressing mutant MACC1-AS1 without a miR-384-5p or miR-145-3p binding site reduced cell proliferation, in contrast to the cells expressing MACC1-AS1. *P *<* *0.05*.

Within the six MACC1-AS1-associated miRNAs, four have been reported to serve as growth-suppressive miRNAs in different cell contexts through regulation of their corresponding mRNA targets. For example, miR-384 targets PTN (pleiotrophin) mRNA to inhibit cell proliferation^23^, and miR-145-3p silences the Warburg effect-promoting proto-oncogene c-Myc mRNA in bladder cancer cells^24^. We found that in cells overexpressing MACC1-AS1, both mRNA and protein levels of PTN and c-Myc were upregulated (Fig. 4a, b). In contrast, expression of PTN or c-Myc mRNA was barely altered in the cells overexpressing MACC1-AS1 lacking either the miR-145-3p (mut-145) or the miR-384 (mut-384) binding site (Fig. 4c, d), indicating a sponge effect of MACC1-AS1 for miR-384-5p and miR-145-3p. In addition, miR-384-mediated TPN or miR-145-3p-mediated c-Myc mRNA reduction could be partially rescued by overexpression of MACC1-AS1 (Fig. 4e). Moreover, binding of MACC1-AS1 to the miRNAs altered the cell growth phenotype, since cells expressing mutant MACC1-AS1 without the miR-384 or miR-145-3p binding site reduced cell proliferation, in comparison to the cells expressing MACC1-AS1 (Fig. 4f). These results suggest a role for MACC1-AS1 in promoting cell proliferation through competitively sponging corresponding miRNAs.

### PTBP1 is a MACC1-AS1-binding partner

**Fig. 5:**
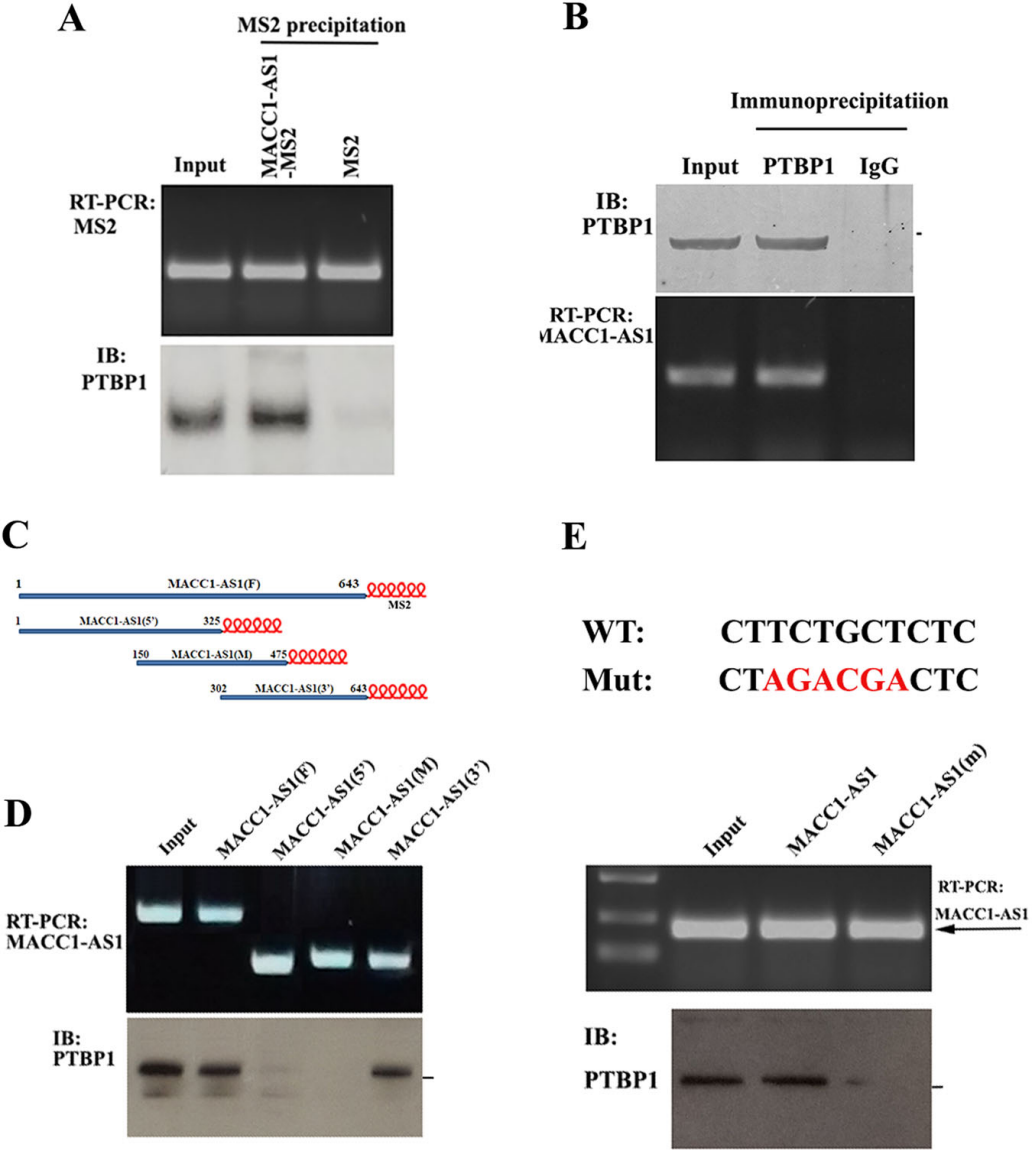
Identification of PTBP1 as a MACC1-AS1-binding partner. **a** PTBP1 co-precipitated with MACC1-AS1. Upper: MACC1-AS1-MS2-pulldown assays, MS2 was used as a control. Lower: western blot analysis of the presence of PTBP1 in the MACC1-AS1-MS2 precipitates. **b** MACC1-AS1 was detected in the PTBP1 IP. Upper: western blots of PTBP1 in the IP, normal IgG was used as a control. Lower: RT-PCR and agarose gel electrophoresis indicated the association of MACC1-AS1 with PTBP1. **c** Schematic representation of truncated MACC1-AS1-MS2 RNAs, which can be bound to amylose resin through MBP-MCP. **d** Upper: vectors expressing MS2-fused full-length MACC1-AS1 or truncated MACC1-AS1 (5′, middle (m) and 3′) were transiently transfected into HEK-293T cells. RT-PCR showing that individual MS2-fused RNAs were pulled down by MBP-MCP attached amylose resin. Lower: western blots indicating that PTBP1 co-precipitated with the full-length and the 3′ part of MACC1-AS1. **e** Upper: a putative motif for the PTBP1 binding site within MACC1-AS1 is shown. Red nucleotides indicates the mutated sequences. Lower; wild-type and mutant MACC1-AS1-MS2 RNAs were transfected into HEK-293T cells. MS2-pulldown assays and immunoblots showed that PTBP1 was barely detected in the PTBP1 precipitates when the PTBP1-binding motif within MACC1-AS1 was mutated.

Growing information indicates that lncRNAs often exert their functions by binding to protein partners. To identify potential proteins that interact with MACC1-AS1, we performed in vitro RNA pulldown assays using in vitro synthesized chimeric MACC1-AS1-MS2 or MS2 RNAs, which were attached to amylose resin by recombinant MBP-MCP. After incubation with cell lysate, MACC1-AS1-attached maltose resin was precipitated, and the proteins associated with MACC1-AS1 were collected for further analysis. MS protein sequencing assays identified a number of proteins bound to MACC1-AS1-MS2, in contrast to control MS2 RNA (MS spectrometry data has been deposited in the ProteomeXchange database as accession number of IPX0001686000). One of them was PTBP1 (Suppl Table 1, and Suppl. Fig. S4), an RBP that has been reported to be involved in RNA regulation^25^. Interaction of MACC1-AS1 with PTBP1 was verified by MS2 pulldown assays in cell extracts expressing MACC1-AS1-MS2 chimeric RNA or MS2 using amylose resin-conjugated MBP-MCP (Fig. 5a). Alternatively, we performed RIP experiments using PTBP1 antibody and detected MACC1-AS1 in the PTBP1 precipitates (Fig. 5b), further confirming that PTBP1 is truly complexed with MACC1-AS1. To determine which part of MACC1-AS1 is involved in the interaction with PTBP1, we dissected MACC1-AS1 into three regions (5′ middle and 3′) by PCR and subcloned them into lentivirus constructs to express MS2 hairpin-tagged truncated MACC1-AS1 RNAs (Fig. 5c, upper panel). After transfection of the individual constructs into HEK-293T cells, MS2-pulldown assays were performed in the cell lysates expressing individual chimeric MACC1-AS1 RNAs. PTBP1 was detected in the precipitates of both full-length and the 3′ region of MACC1-AS1, whereas the 5′ and the middle region (M) of MACC1-AS1 did not provide a visible signal (Fig. 5d, lower panel). Since PTBP1 has been previously reported to bind to a pyrimidinerich region of RNA^26^, we searched the nucleotide sequence of MACC1-AS1 and found a sequence 5′-CTTCTGCTCTC-3′ at the 3′ region of MACC1-AS1 (478-488, Suppl Fig. S2A), which is homologous to the conserved PTBP1-binding motif 5′-CYYYYCYYYY(Y/G)G -3′, where Y represents C or T. We than mutated this binding motif and transfected the mutant (MACC1-AS1(m)) into cells. RNA pulldown assays indicated that mutation of the putative binding site within MACC1-AS1 dramatically reduced the binding of PTBP1 to MACC1-AS1 (Fig. 5e), suggesting that this motif is responsible for PTBP1 binding.

### PTBP1 stabilizes MACC1-AS1 and the binding of MACC1-AS1 to miRNAs

**Fig. 6:**
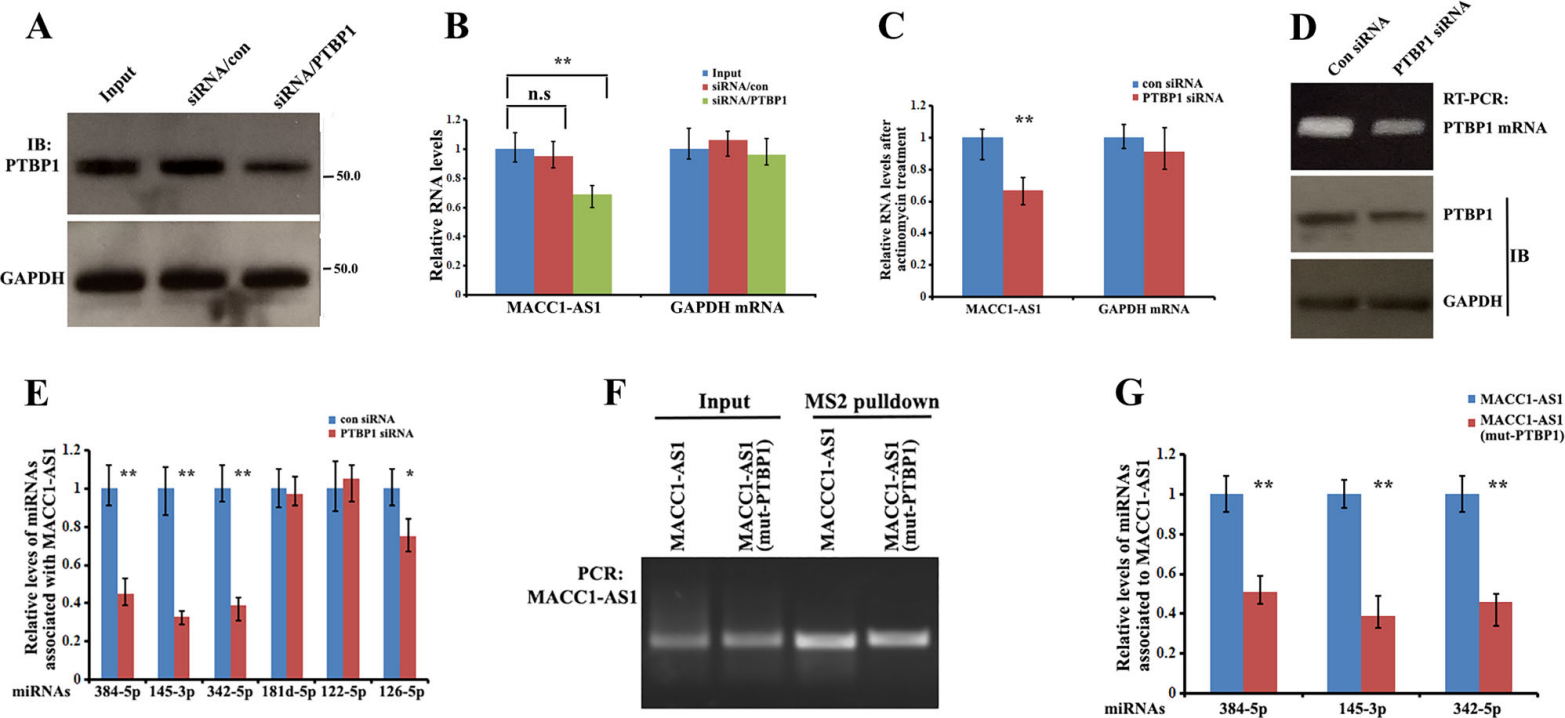
PTBP1 stabilizes MACC1-AS1 and the binding ability of MACC1-AS1 to miRNAs. **a** Western blots showing the expression of PTBP1 in BT549 cells after transfection with PTBP1 siRNA or scrambled siRNA for 48 h. **b** qRT-PCR indicating the relative levels of MACC1-AS1 in PTBP1 knockdown and control cells. Data are presented as means ± SD from three independent experiments. **P *<* *0.01* as determined by one-way ANOVA assay. **c** Levels of MACC1-AS1 in BT549 and BT549/PTBP1-siRNA cells were analyzed after treatment with actinomycin D for 12 h. Relative MACC1-AS1 levels were determined by qRT-PCR. Data are presented as means ± SD from three independent experiments. **P *<* *0.01* as determined by Student’s *t* test. **d** MDA-MB-231/MACC1-AS1-MS2 cells were transfected with PTBP1 siRNA for 48 h. RNA agarose gel electrophoresis and western blots indicated downregulation of PTBP1 mRNA and protein. GAPDH was used as a control. **e** Cells transfected as in (**d**) were subjected qRT-PCR to detect the levels of individual miRNAs associated with MACC1-AS1 in pulldown precipitates. **P *<* *0.01* and *P *<* *0.05* as determined by Student’s *t* test. **f** MDA-MB-231 cells were transfected with constructs expressing MACC1-AS1-MS2 and MACC1-AS1(m)-MS2 for 72 h. RNA agarose gel electrophoresis showed the precipitated MACC1-AS1-MS2 and MACC1-AS1(m)-MS2 in cell extracts. **g** Cells transfected as in (**f**) were subjected qRT-PCR, showing that levels of MACC1-AS1-associated miRNAs were greatly reduced when the biding site for PTBP1 was mutated. **P *<* *0.01* as determined by Student’s *t* test.

To determine the biological consequence of PTBP1 and MACC1-AS1 interaction, we examined the effect of PTBP1 on MACC1-AS1 expression in BT549 cells. RT-qPCR analysis from total RNA revealed that silencing of PTBP1 expression by siRNA significantly reduced MACC1-AS1 steady-state levels (Fig. 6a, b). To study if this reduction was due to increased MACC1-AS1 decay, cells were cultured in the presence of actinomycin D that blocks transcription. Compared to control cells, knockdown of PTBP1 reduced MACC1-AS1 levels to about 50% after 24 h incubation with actinomycin D (Fig. 6c). These results indicate that binding of PTBP1 decreases the decay of MACC1-AS1.

Next, we investigated whether PTBP1 affects the binding of miRNAs to MACC1-AS1. We performed MS2 pulldown assays in MACC1-AS1-MS2-expressing MDA-MB-231 cells transfected with PTBP1 siRNA (Fig. 6d). Results showed that knockdown of PTBP1 considerably decreased the binding of miR-145-3p, miR-384, or miR-342-5p to MACC1-AS1 (Fig. 6e). However, association of MACC1-AS1 with other miRNAs, miR-122-5p or miR-181d-5p was not significantly changed (Fig. 6e). In contrast, in cells expressing a mutant MACC1-AS1, whose putative binding site for PTBP1 is mutated, the ability of MACC1-AS1 to sponge miR-145-3p or miR-384 was reduced (Fig. 6f, g). These results suggest that binding of PTBP1 not only stabilizes MACC1-AS1, but also selectively enhances the sponge effect of MACC1-AS1 to individual miRNAs.

### Competitive binding of MACC1-AS1 to PTBP1 affects PTBP1 target mRNA fate

**Fig. 7:**
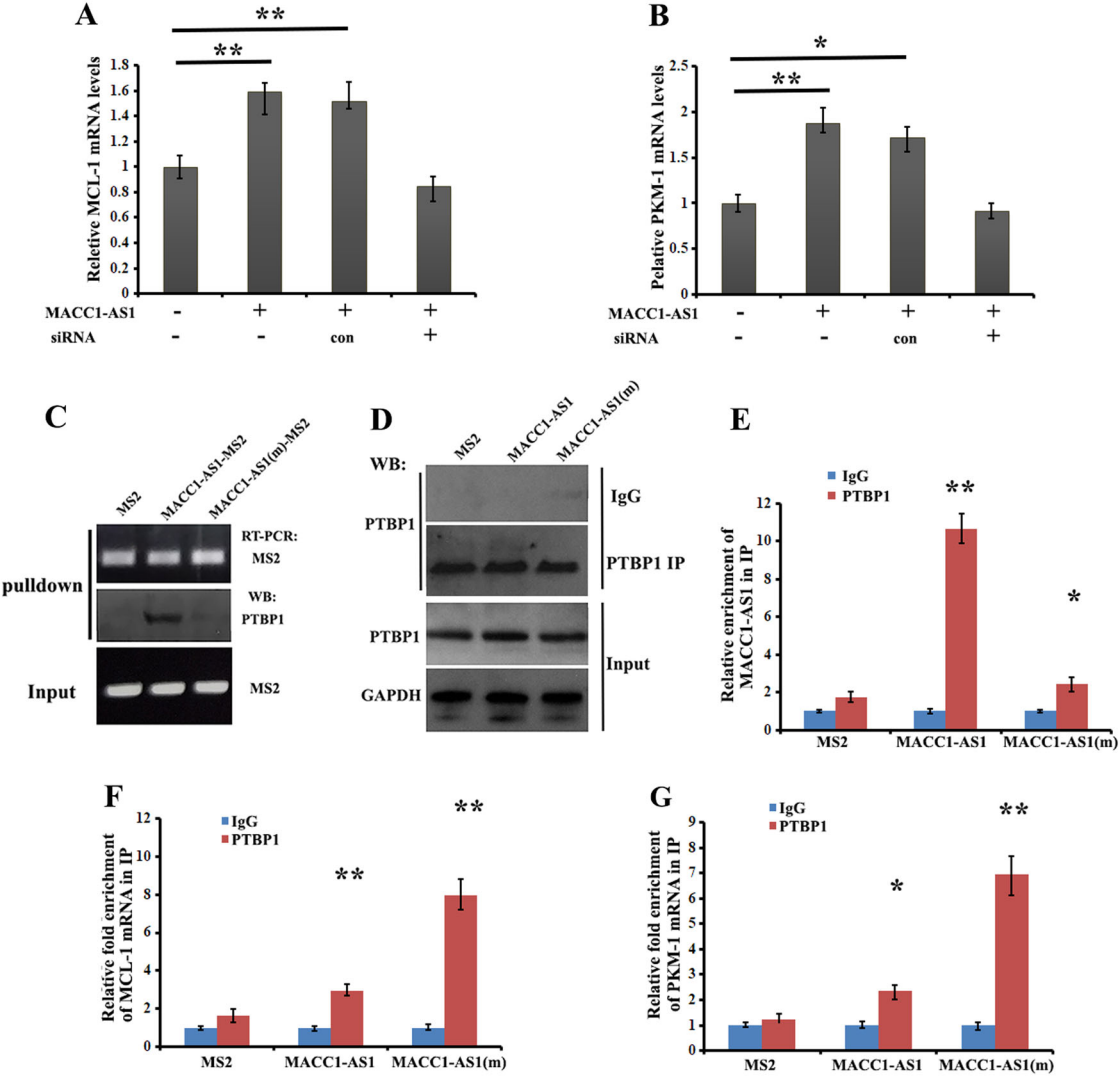
Competitive binding of MACC1-AS1 to PTBP1 affects PTBP1 target mRNA fate. **a**, **b** qRT-PCR was performed to assess the levels of MCL-1 and PKM-1 mRNAs in MACC1-AS1-expressing MDA-MB-231 cells and in cells expressing siRNA against MACC1-AS1. **P *<* *0.01* and *P *<* *0.05* as determined by Student’s *t* test. **c** MDA-MB-231 cells transfected with constructs expressing MS2, MACC1-AS1-MS2 or MACC1-AS1(m)-MS2 for 72 h were subjected to MS2 pulldown assays. Representative western blots indicate that MACC1-AS1(m) was unable to associate with PTBP1. **d** Cells transfected as in (**c**) were subjected to immunoprecipitation with PTBP1 antibody or control IgG. Precipitated PTBP1 is shown by western blots. GAPDH was used as a control. **e** Levels of MACC1-AS1 and MACC1-AS1(m) in the PTBP1 precipitates were measured by qRT-PCR. MACC1-AS1(m) was not able to co-precipitate with PTBP1, when the binding site for PTBP1 was mutated. **P *<* *0.01* as determined by Student’s *t* test. **f**, **g** Levels of MCL-1 and PKM-1 mRNAs in the PTBP1 immunoprecipitates as in (**d**) were analyzed by qRT-PCR. Binding of MCL-1 and PKM-1 mRNAs to PTBP1 was significantly reduced in cells overexpressing MACC1-AS1. **P *<* *0.01* and *P *<* *0.05* as determined by Student’s *t* test.

It has been reported in many studies that PTBP1 can regulate post-transcriptional expression of genes through controlling their stability and translation^27,28^. Based on the fact that MACC1-AS1 is able to bind PTBP1, we hypothesized that the target mRNAs of PTBP1 could be differentially influenced by MACC1-AS1 expression. To address this, we tested the effect of MACC1-AS1 on the expression of MCL-1 (myeloid cell leukemia protein-1) and PKM-1 (pyruvate kinase) mRNAs, both of which should be destabilized by binding to PTBP1^29,30^. Levels of MCL-1 and PKM-1 mRNAs were significantly upregulated in MACC1-AS1-overexpressing MDA-MB-231 cells; however, increased expression of MCL-1 and PKM-1 mRNAs could be downregulated when MACC1-AS1 was silenced by siRNA treatment (Fig. 7a, b). We next transfected MDA-MB-231 cells with constructs expressing MS2, MACC1-AS1-MS2 or MACC1-AS1(m)-MS2 for 72 h. Using the cell extracts, we performed MS2 pulldown and western blots and confirmed that PTBP1 was co-precipitated with MACC1-AS1, but not with MACC1-AS1(m), which lacked the biding site for PTBP1 (Fig. 7c). We then performed immunoprecipitation using PTBP1 antibody or control IgG (Fig. 7d), and analyzed the levels of MACC1-AS1, MCL-1 and PKM-1 mRNAs by qRT-PCR in the IP precipitates, represented as fold enrichment in PTBP1 IP relative to control IgG IP. MACC1-AS1 was highly enriched in the precipitates of PTBP1 antibody (Fig. 7e). However, levels of both MCL-1 and PKM-1 mRNAs in the precipitates of PTBP1 antibody were reduced when MACC1-AS1 was overexpressed, compared to cells expressing empty MS2 (a construct that does not contain MACC1-AS1 segments). In comparison, levels of MCL-1 and PKM-1 mRNAs in MACC1-AS1(m)-overexpressing cells were not obviously changed, indicating the preferential binding of PTBP1 to MACC1-AS1 (Fig. 7f, g). Taken together, these experiments suggest that the increased expression of MCL-1 and PKM-1 mRNAs in MACC1-AS1-overexpressing cells resulted from the shift of PTBP1 to MACC1-AS1 RNA and that blocking the binding of MACC1-AS1 to PTBP1 restores the protein availability to its target MCL-1 and PKM-1 mRNAs.

**Fig. 8:**
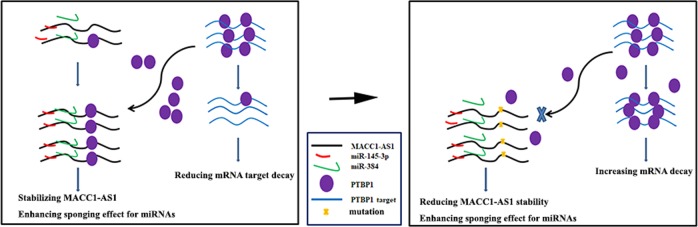
A model of MACC1-AS1 sponges miRNAs and PTBP1. **a** MACC1-AS1 sponges multiple miRNAs, preventing their interaction with target mRNAs. Meanwhile, competitive binding of MACC1-AS1 to PTBP1 stabilizing this RNA and enhances the sponge effect of MACC1-AS1 to miRNAs. **b** When binding of MACC1-AS1 to PTBP1 is impaired due to mutation, PTBP1 becomes available to target mRNAs, stabilizing the mRNAs and promoting their expression.

Based on our findings, we predict a model where competitive binding of PTBP1 to MACC1-AS1 not only stabilizes this RNA and enhances the sponge effect of MACC1-AS1 to multiple miRNAs, but also traps PTBP1 away from its target mRNAs. A MACC1-AS1 mutant with impaired ability to bind PTBP1 is unable to associate with PTBP1, permitting PTBP1 to bind to its target mRNAs and elicit its post-transcriptional function (Fig. 8).

## Discussion

Accumulating evidence indicates that lncRNAs play important roles in cancer biology. However, only a few lncRNAs have been functionally and mechanically characterized. In the current study, we demonstrate that ectopic expression of MACC1-AS1 promotes cell proliferation and breast tumor progression. We show that MACC1-AS1-mediated tumorigenesis occurs at least in part through the interaction with multiple miRNAs. Specifically, MACC1-AS1 sponges miR-384 and miR-145-3p to reduce their activity to PTN and c-Myc mRNAs. We further revealed a regulation that PTBP1 physically interacts with MACC1-AS1. This interaction not only stabilizes this lncRNA and increases the sponge effect of MACC1-AS1 to miRNAs, but also sequesters PTBP1 availability away from its target mRNAs.

Antisense lncRNAs are able to bind to corresponding mRNAs and regulate their expression post-transcriptionally^22^. MACC1-AS1 has been shown to induce gastric cancer cell metabolic plasticity through upregulation MACC1 mRNA^20^, which is an important player in promoting proliferation and invasion of variety of cancer cells^18,19^. Although we observed that MACC1-AS1-mediated breast cancer progression is associated with increased expression of MACC1 mRNA, we did not detect direct interaction of MACC1-AS1 with MACC1 mRNA in vivo (Fig. 2). This indicates that an additional mechanism is involved in regulating MACC1 gene expression and this mechanism is currently under investigation. Since nuclear/cytoplasmic fractionation and immunoprecipitation assays showed that MACC1-AS1 is also widely distributed in the cytoplasm and complexed with Ago2, we hypothesized that MACC1-AS1 could have a cytoplasmic function serving as a platform for miRNAs. Indeed, we identified six miRNAs to competitively interact with MACC1-AS1, some of which, such as miR-145-3p and miR-384, have high binding affinity (Fig. 3). Such a sponge effect would allow MACC1-AS1 to modulate cell growth through regulating the expression of the miRNA targets (Fig. 4). Thus, our study reveals an unusual mechanism whereby a lncRNA could trigger an oncogenic role by serving as a binding platform for multiple miRNAs.

LncRNAs have been shown to play a wide range of biological roles through diverse molecular mechanisms often including the interaction with one or more protein partners^31^. Using the method described in the study, termed MS2-tagged RNA affinity purification, we isolate MACC1-AS1 RNA complexes and analyzed putative protein factors associated with MACC1-AS1 in the context of the intact cell. As a result of the assay, an RBP, PTBP1, involved in the regulation of various RNAs^32^, has been identified and shown to bind to the conserved region of MACC1-AS1. We found that binding of PTBP1 not only stabilizes MACC1-AS1, but also enhances the ability of MACC1-AS1 to sponge miR-145-3p and miR-384 miRNAs (Fig. 5 and Fig. 6). Interestingly, miR-145-3p indirectly induces downregulation of PTBP1 transcription through repressing c-Myc expression^24^, suggesting a positive feedback loop between MACC1-AS1 and PTBP1 interaction. Since MS2-tagged RNA affinity purification identified a group of RBPs that complex with MACC1-AS1 (e.g. DDX5, HNRNPK and HNRNPA1), it will be interesting to investigate whether they are also involved in the function of regulation of MACC1-AS1.

LncRNAs are able to serve as sponges either for miRNAs or for RBPs by functioning as a competing endogenous RNA^33,34^. For instance, lncRNA UCA1 has been shown to sponge miRNA miR-122-5p^7^ and lncRNA H19 sponges miRNAs miR-138 and miR-200a;^35^ while lncRNA OIP5-AS1 has an ability to sponge RBP HuR from its target mRNAs^8^. In the present study, we determine that MACC1-AS1 functions as an endogenous competing RNA for both miRNAs and RNA-biding proteins. Our findings show that impairment of the binding ability of MACC1-AS1 for miR-384 or miR-145-3p affects their target expression (PTN and c-Myc), resulting in decreased cell proliferation (Fig.3 and Fig.4). Moreover, MACC1-AS1 has also shown the capability of preferentially binding to PTBP1 (Fig. 7). Prevention of PTBP1-MACC1-AS1 interaction destabilizes MACC1-AS1 itself and lowers the sponge effect of MACC1-AS1 for miRNAs (Fig. 5 and Fig. 6). Based on these results, it seems that in breast cancer cells, the oncogenic function of MACC1-AS1 largely relies on coordinated association of MACC1-AS1 with PTBP1 and individual miRNAs. Altering the sponge effect of MACC1-AS1 for PTBP1 and the miRNAs could change the expression of many genes and eventually affect cell proliferation and invasion.

In conclusion, our findings reveal that PTBP1 and multiple miRNAs coordinately interacts with MACC1-AS1 and that the net impact of the PTBP1-MACC1-AS1 interaction influences the pattern of MACC1-AS1-bound miRNAs, and hence the concentration of miRNAs available for their target mRNAs. These data support the notion that MACC1-AS1 can be a binding platform for target miRNAs and PTBP1, and the binding efficiency eventually modulates the post-transcriptional fate of genes targeted by the miRNAs and PTBP1.

## Supplementary information

Legends of suppl information

Supp Figure S1

Supp Figure S2

Supp Figure S3

Supp Figure S4

Supp table S1

Supp table S2

Supp table S3

## Data Availability

All data generated or analyzed during this study are included in this published article (and its supplementary information files).

## References

[1] Kung JT, Colognori D, Lee JT (2013). Long noncoding RNAs: past, present, and future. Genetics.

[2] Yoon JH, Abdelmohsen K, Gorospe M (2013). Posttranscriptional gene regulation by long noncoding RNA. J. Mol. Biol..

[3] Gupta RA (2010). Long non-coding RNA HOTAIR reprograms chromatin state to promote cancer metastasis. Nature.

[4] Yoon JH (2013). Scaffold function of long non-coding RNA HOTAIR in protein ubiquitination. Nat. Commun..

[5] West JA (2014). The long noncoding RNAs NEAT1 and MALAT1 bind active chromatin sites. Mol. cell..

[6] Chen S (2017). The lncRNA HULC functions as an oncogene by targeting ATG7 and ITGB1 in epithelial ovarian carcinoma. Cell Death Dis..

[7] Zhou Y (2018). IMP1 regulates UCA1-mediated cell invasion through facilitating UCA1 decay and decreasing the sponge effect of UCA1 for miR-122-5p. Breast Cancer Res.: Bcr..

[8] Kim J (2016). LncRNA OIP5-AS1/cyrano sponges RNA-binding protein HuR. Nucleic Acids Res..

[9] Liu X, Li D, Zhang W, Guo M, Zhan Q (2012). Long non-coding RNA gadd7 interacts with TDP-43 and regulates Cdk6 mRNA decay. EMBO J..

[10] Wang X, Vukovic L, Koh HR, Schulten K, Myong S (2015). Dynamic profiling of double-stranded RNA binding proteins. Nucleic Acids Res..

[11] Hua F, Li K, Yu JJ, Hu ZW (2015). The TRIB3-SQSTM1 interaction mediates metabolic stress-promoted tumorigenesis and progression via suppressing autophagic and proteasomal degradation. Autophagy.

[12] Wilusz JE, Sunwoo H, Spector DL (2009). Long noncoding RNAs: functional surprises from the RNA world. Genes &. Development.

[13] Castello A (2012). Insights into RNA biology from an atlas of mammalian mRNA-binding proteins. Cell.

[14] Keene JD (2007). RNA regulons: coordination of post-transcriptional events. Nat. Rev. Genet..

[15] Ferre F, Colantoni A, Helmer-Citterich M (2016). Revealing protein-lncRNA interaction. Brief. Bioinforma..

[16] Huntzinger E, Izaurralde E (2011). Gene silencing by microRNAs: contributions of translational repression and mRNA decay. Nat. Rev. Genet..

[17] Fabian MR, Sonenberg N, Filipowicz W (2010). Regulation of mRNA translation and stability by microRNAs. Annu. Rev. Biochem..

[18] Shirahata A (2011). MACC 1 as a marker for vascular invasive hepatocellular carcinoma. Anticancer Res..

[19] Shirahata A (2010). MACC 1 as a marker for peritoneal-disseminated gastric carcinoma. Anticancer Res..

[20] Zhao Y (2018). The lncRNA MACC1-AS1 promotes gastric cancer cell metabolic plasticity via AMPK/Lin28 mediated mRNA stability of MACC1. Mol. Cancer.

[21] Song T (2015). Specific interaction of KIF11 with ZBP1 regulates the transport of beta-actin mRNA and cell motility. J. Cell Sci..

[22] Carrieri C (2012). Long non-coding antisense RNA controls Uchl1 translation through an embedded SINEB2 repeat. Nature.

[23] Bai PS, Xia N, Sun H, Kong Y (2017). Pleiotrophin, a target of miR-384, promotes proliferation, metastasis and lipogenesis in HBV-related hepatocellular carcinoma. J. Cell. Mol. Med..

[24] Takai T (2017). A novel combination RNAi toward Warburg effect by replacement with miR-145 and silencing of PTBP1 induces apoptotic cell death in bladder cancer cells. Int. J. Mol. Sci..

[25] Cui J, Placzek WJ (2018). PTBP1 enhances miR-101-guided AGO2 targeting to MCL1 and promotes miR-101-induced apoptosis. Cell Death Dis..

[26] Tillmar L, Carlsson C, Welsh N (2002). Control of insulin mRNA stability in rat pancreatic islets. Regulatory role of a 3'-untranslated region pyrimidine-rich sequence. J. Biol. Chem..

[27] Sawicka K, Bushell M, Spriggs KA, Willis AE (2008). Polypyrimidine-tract-binding protein: a multifunctional RNA-binding protein. Biochemical Soc. Trans..

[28] Xue Y (2013). Direct conversion of fibroblasts to neurons by reprogramming PTB-regulated microRNA circuits. Cell.

[29] Cui J, Placzek WJ (2016). PTBP1 modulation of MCL1 expression regulates cellular apoptosis induced by antitubulin chemotherapeutics. Cell Death Differ..

[30] He X (2014). Involvement of polypyrimidine tract-binding protein (PTBP1) in maintaining breast cancer cell growth and malignant properties. Oncogenesis.

[31] Mohamadkhani A (2014). Long Noncoding RNAs in Interaction With RNA Binding Proteins in Hepatocellular Carcinoma. Hepat. Monthly..

[32] Ge Z, Quek BL, Beemon KL, Hogg JR (2016). Polypyrimidine tract binding protein 1 protects mRNAs from recognition by the nonsense-mediated mRNA decay pathway. eLife.

[33] Cesana M (2011). A long noncoding RNA controls muscle differentiation by functioning as a competing endogenous RNA. Cell.

[34] Ebert MS, Neilson JR, Sharp PA (2007). MicroRNA sponges: competitive inhibitors of small RNAs in mammalian cells. Nat. Methods.

[35] Liang WC (2015). The lncRNA H19 promotes epithelial to mesenchymal transition by functioning as miRNA sponges in colorectal cancer. Oncotarget.

