# Supplementary material for: LncRNA MACC1-AS1 sponges multiple miRNAs and RNA-binding protein PTBP1
Source: Oncogenesis. 2019 Dec 10;8(12):73. doi: 10.1038/s41389-019-0182-7 (PMC6904680; doi:10.1038/s41389-019-0182-7)
Supplement: Supplementary file 1 — Legends of suppl information [file 41389_2019_182_MOESM1_ESM.docx]

MACC1-AS1 regulates cancer cell progression by sponging multiple miRNAs and PTBP1

Xiaona Zhang§, Yanchun Zhou§, Shaoying Chen, Wei Li, Weibing Chen and Wei Gu^1^*

Legends of suppl information

Suppl Figure S1 MACC1-AS1 did not interact with primary MACC1 mRNA

(A) MS2-fused MACC1-AS1 RNA was pulled down by MBP-MCP. qRT-PCR indicated that MACC1-AS1 did not co-precipitate with the sixth intron of primary MACC1 mRNA. (B) FISH (fluorescence in situ hybridization) experiments showed that MACC1-AS1 transcript was widely localized in both nucleus and cytoplasm.

Suppl Figure S2 MACC1-AS1 containing multiple miRNA binding sides

(A) The nucleotide sequence of MACC1-AS1 is shown. Colored sequences indicate the putative binding sites for miRNAs. The underlined sequences indicate the putative binding motif for PTBP1. (B) Six putative miRNA binding site within the MACC1-AS1 sequence was mutated to assess the interactions between MACC1-AS1 and individual miRNAs. Red sequences indicate the mutant nucleotides on each miRNA binding site.

Suppl Figure S3 binding of individual miRNAs does not affect endogenous MACC1-AS1 expression

Individual miRNAs were transfected into BT549 cells. qRT-PCR indicated that the levels of endogenous MACC1-AS1 were not significantly reduced.

Suppl Figure S4 Identification of PTBP1 as a MACC1-AS1 binding protein

Mass spectrometric analysis of the proteins precipitated with MACC1-AS1 identified PTBP1. The coverage rate of the seven peptides sequences reaches 29% for PTBP1 with 100% matching. Highlighted sequences in the figure show the peptides that completely match the sequences of PTBP1.

Suppl Table S1

Proteins co-precipitated with MACC1-AS1 were analyzed by MS spectrometric assays. TPBP1 is one of the proteins identified to be associated with PTBP1.

Suppl Table S2

Nucleotide sequences of the primers used to make MACC1-AS1 constructs and MACC1-AS1 mutants are shown. The primers were mainly used for analyzing interactions of MACC1-AS1 with individual miRNAs and PTBP1.

Suppl Table S3

The table listed nucleotide sequences for detecting the expression of MACC1-AS1, MACC1, PTBP1, c-Myc and GAPDH RNAs by qRT-PCR. The nucleotide sequences of miRNA mimics and siRNAs were used for knockdown gene expression in experiments.
