# Supplementary figures and images for: LncRNA MACC1-AS1 sponges multiple miRNAs and RNA-binding protein PTBP1
Source: Oncogenesis. 2019 Dec 10;8(12):73. doi: 10.1038/s41389-019-0182-7 (PMC6904680; doi:10.1038/s41389-019-0182-7)

Suppl Fig. S1

**A**

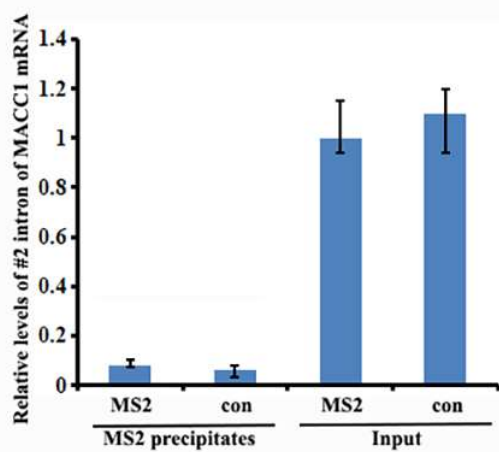

**B**

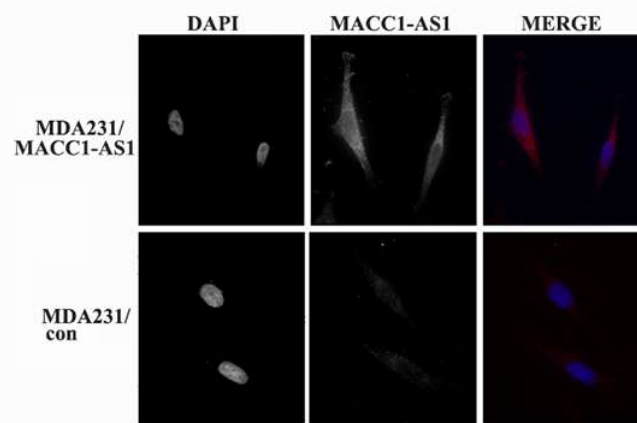

Supplement: Supplementary file 2 — Supp Figure S1 [file 41389_2019_182_MOESM2_ESM.pdf]

Suppl Fig. S3

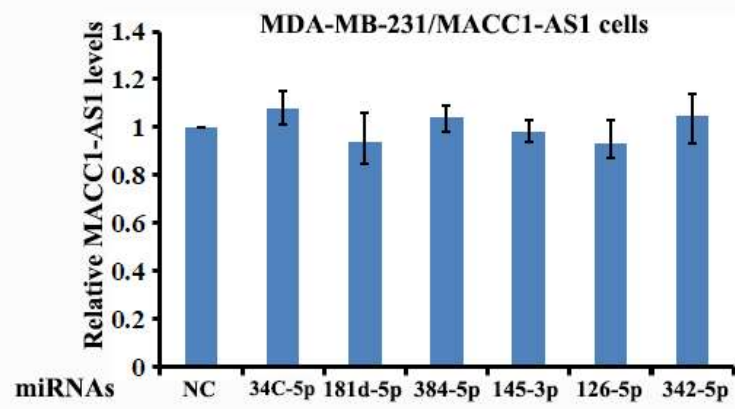

Supplement: Supplementary file 4 — Supp Figure S3 [file 41389_2019_182_MOESM4_ESM.pdf]
