# Supplementary material for: LncRNA MACC1-AS1 sponges multiple miRNAs and RNA-binding protein PTBP1
Source: Oncogenesis. 2019 Dec 10;8(12):73. doi: 10.1038/s41389-019-0182-7 (PMC6904680; doi:10.1038/s41389-019-0182-7)
Supplement: Supplementary file 3 — Supp Figure S2 [file 41389_2019_182_MOESM3_ESM.pdf]

Suppl Fig. S2

A

```
                               miR-181d-5p
1    CAAATTGTAGAATACACACACACACGCACACACACACACACGTCACACACACAGCACCTT
61   AAAATGAATTTTCTCTAGGGAAACAACAAAATGAACCCTGCACTTGAACAACACTTCATT
              miR-34C-5p                     miR-126-5p
121  TTCAGTACCATTAGCTGAAGAAGTATTAAGGCAAGGTTGCCTTCTTAATGATTATTTCAT
181  GTGTTCTATCCTCAGTACATCATTTACAATATTCAGACTTGGAACAAAGTACTTTGAACT
241  CTCCAGCGGCCAGTCAGAAAATGAGGAACAGGTTGTGAATAGAAGATACAAAGGAGTATT
301  AGATCTGCATTGATTAAATGGTGACTACAAATAGACTTAAAATACATCTTCCCAATTATG
         mir-342-5p                     mir-145-3p
361  AATCTCTACAGGTGGTTTTGTCCTGTTCACCCAACTGGAATGGTTGCATCGAAGCAAGAA
         miR-384-5p
421  AATTTTCTCCAAAAATTAATACCCAGATCTACGCCAAAAACAAAGTGAAGAATACAGCTT
481  CTGCTCTCACCAAGTCATCTCTAATAGAGAGAAGATTTCCTTAAACATAAATATCTTCAA
541  AATGCTTGTTCATTTGAGAGAAAGATACAGAATATTTTACATGTGCCCTTCACTGGAATA
601  TTTTTATTTATTTAATTCAATAAAAATTGACTCCCAGTT
```

B

miR-181d-5p(50-72)	WT	5′	CACAGCACCTTAAAATGAATTTT	3′
	Mut		CACAGCACCTTAATTACTTATTT	
miR-34C-5p(127-147)	WT	5′	ACCATTAGCTGAAGAAGTATTA	3′
	Mut		ACCTAGTCGAGAAGAAGTATTA	
miR-126-5p(150-173)	WT	5′	GGCAAGGTTGCCTTCTTAATGATT	3′
	Mut		GGCAAGGTTGCCTTCTATTATTAT	
miR-342-5p(354-374)	WT	5′	AATTATGAATCTCTACAGGTG	3′
	Mut		AATTATGAATCTCTTGTCTAG	
miR-145-3p(387-406)	WT	5′	GTTCACCCAACTGGAATGG	3′
	Mut		GTGGTGGGTACTGGAATGG	
miR-384-5p(417-437)	WT	5′	AGAAAATTTTCTCCAAAAATT	3′
	Mut		AGAAATAAAGATCCAAAAATT	
