# Supplementary material for: LncRNA MACC1-AS1 sponges multiple miRNAs and RNA-binding protein PTBP1
Source: Oncogenesis. 2019 Dec 10;8(12):73. doi: 10.1038/s41389-019-0182-7 (PMC6904680; doi:10.1038/s41389-019-0182-7)
Supplement: Supplementary file 5 — Supp Figure S4 [file 41389_2019_182_MOESM5_ESM.pdf]

Suppl Fig. S4

1 **MDGIVPDIAV GTK**RGSDELF STCVTNGPFI MSSNSASAAN GNDSKKFKGD SRSAGVPSRV IHIR**KLPIDV TEGEVISLGL**

81 **PFGK**VTNLLM LKGKNQAFIE MNTEEAANTM VNYYTSVTPV LR**GQPIYIQF SNHK**ELKTDS SPNQAR**AQAA LQAVNSVQSG**

161 **NLALAASAAA VDAGMAMAGQ SPVLR**IIVEN LFYPVTLDVL HQIFSKFGTV LKIITFTK**NN QFQALLQYAD PVSAQHAK**LS

241 LDGQNIYNAC CTLRIDFSKL TSLNVKYNND KSRDYTRPDL PSGDSQPSLD QTMAAAFGLS VPNVHGALAP LAIPSAAAAA

321 AAAGR**IAIPG LAGAGNSVLL VSNLNPER**VT PQSLFILFGV YGDVQRVKIL FNKK**ENALVQ MADGNQAQLA MSHLNGHK**LH

401 GKPIRITLSK HQNVQLPREG QEDQGLTKDY GNSPLHRFKK PGSKNFQNIF PPSATLHLSN IPPSVSEEDL KVLFSSNGGV

481 VKGFKFFQKD RKMALIQMGS VEEAVQALID LHNHDLGENH HLRVSFSKST I

Mass spectrometric analysis of the protein precipitated with MACC1-AS1 identified PTBP1. The coverage rate of the seven peptide sequence reached 29% for PTBP1 with 100% maching. Highlited sequences in the figure show the peptides that complementely mached the sequence of the PTBP1 protein.
