# Supplementary material for: LncRNA MACC1-AS1 sponges multiple miRNAs and RNA-binding protein PTBP1
Source: Oncogenesis. 2019 Dec 10;8(12):73. doi: 10.1038/s41389-019-0182-7 (PMC6904680; doi:10.1038/s41389-019-0182-7)
Supplement: Supplementary file 6 — Supp table S1 [file 41389_2019_182_MOESM6_ESM.pdf]

Suppl Table S1 List of MACC1-AS1 interacting proteins identified by MS analysis

Protein name	Mass (Da)	Area in vitro MACC1-AS1-MS2	-10lgP	Peptides	Unique
PTBP1	57221	1.46E+06	188.18	7	7
SYNCRIP	69603	1.28E+06	268.1	16	15
ILF2	43062	8.66E+04	252.99	13	13
DDX5	69148	3.95E+05	148.07	7	5
HNRNPL	64133	3.07E+05	192.87	6	6
HNRNPD	38434	5.96E+05	184.3	6	5
HNRNPAB	36225	2.98E+05	164.44	5	5
HNRNPA2B1	37430	1.53E+05	157.93	5	3
PABPC1	70671	1.04E+05	155.6	6	3
HNRNPUL2	85105	3.45E+05	146.02	6	6
MYBBP1A	148854	6.44E+04	145.64	4	4
HNRNPA0	30841	4.75E+05	145.27	3	3
IGF2BP2	66121	5.95E+04	143.16	5	3
