# Supplementary material for: LncRNA MACC1-AS1 sponges multiple miRNAs and RNA-binding protein PTBP1
Source: Oncogenesis. 2019 Dec 10;8(12):73. doi: 10.1038/s41389-019-0182-7 (PMC6904680; doi:10.1038/s41389-019-0182-7)
Supplement: Supplementary file 7 — Supp table S2 [file 41389_2019_182_MOESM7_ESM.pdf]

Suppl Table 2

**Primers for MACC1-AS1 and mutants**	**Nucleotide Sequences (red sequences as mutated sequence)**
(XhoI)--MACC1-AS1-F	ccgCTCGAGCAAATTGTAGAATACACACACAC
(NotI)--MACC1-AS1-R	GCGGCCGCAACTGGGAGTCAATTTTTATTGAA
miR-181d-5p mut -F	tacaggtggttttgtcctgtggtgggtactggaatggttgcatcgaag
miR-181d-5p mut -R	cttcgatgcaaccattccagtacccaccacaggacaaaaccacctgta
miR-126d-5p mut -F	ggcaaggttgccttctattattatatttcatgtgttctatcctc
miR-126d-5p mut -R	gaggatagaacacatgaaatataataatagaaggcaaccttgcc
miR-342-5p mut -F	cttcccaattatgaatctcttgtctatgttttgtcctgttcacccaactgg
miR-342-5p mut -R	ccagttgggtgaacaggacaaaacatagacaagagattcataattgggaag
miR-34C-5p mut -F	caacacttcattttcagtacctagtcgagaagaagtattaaggcaaggttgcc
miR-34C-5p mut -R	ggcaaccttgccttaatacttcttctcgactaggtactgaaaatgaagtgttg
miR-384-5p mut -F	catcttcccaattatgataaagatcaggtggttttgtcctgttcaccc
miR-384-5p mut -R	gggtgaacaggacaaaaccacctgatctttatcataattgggaagatg
miR-145-3p mut -F	tacaggtggttttgtcctgtggtgggtactggaatggttgcatcgaag
miR-145-3p mut -R	cttcgatgcaaccattccagtacccaccacaggacaaaaccacctgta
PTBP1-binding site mut-F	caaagtgaagaatacagctagacgactcaccaagtcatctc
PTBP1-binding site mut-R	gagatgacttggtgagtcgtctagctgtattcttcactttg

