# Supplementary material for: LncRNA MACC1-AS1 sponges multiple miRNAs and RNA-binding protein PTBP1
Source: Oncogenesis. 2019 Dec 10;8(12):73. doi: 10.1038/s41389-019-0182-7 (PMC6904680; doi:10.1038/s41389-019-0182-7)
Supplement: Supplementary file 8 — Supp table S3 [file 41389_2019_182_MOESM8_ESM.pdf]

Suppl Table 3   Primers for RT-PCR, miRNA mimics and siRNAs

Primers for real-time PCR	Nucleotide Sequences
MACC1-AS1-F	GAACCCTGCACTTGAACAACAC
MACC1-AS1-R	CTATTCACAACCTGTTCCTCAT
GAPDH-F	GAGTCAACGGATTTGGTCGT
GAPDH-R	TGGGATTTCCATTGATGACA
MACC1-F	GCCAAGAGTTAGTCGCACGTC
MACC1-R	ATCATAGGCAGGTTTCCACATCA
PTBP1-F	AGCGCGTGAAGATCCTGTTC
PTBP1-R	CAGGGGTGAGTTGCCGTAG
C-Myc-F	GGCTCCTGGCAAAAGGTCA
C-Myc-R	CTGCGTAGTTGTGCTGATGT
miRNA mimics & inhibitor	Sequence (GenePharma)
hsa-miR-181d-5p	AACAUUCAUUGUUGUCGGUGGGU
hsa-miR-126-5p	CAUUAUUACUUUUGGUACGCG
hsa-miR-342-5p	AGGGGUGCUAUCUGUGAUUGA
hsa-miR-34C-5p	AGGCAGUGUAGUUAGCUGAUUGC
hsa-miR-384-5p	AUUCCUAGAAAUUGUUCAUA
hsa-miR-145-3p	GGAUUCCUGGAAAUACUGUUCU
hsa-miR145-3p inhibitor	AGAACAGUAUUUCCAGGAAUCC
siRNAs	Sequence (GenePharma)
For PTBP1-1	CAGUUUACCUGUUUUUAAAtt
For PTBP1-2	GCAUCACGCUCUCGAAGCAtt
For MACC1-AS1-1	CACUUGAACAACACUUCAUTT
For MACC1-AS1-2	GAACAAAGUACUUUGAACUTT
For MACC1-AS1-3	GUGCCCUUCACUGGAAUAUTT
